# Diaqua­bis­(5-carb­oxy-2-ethyl-1*H*-imidazole-5-carboxyl­ato-κ^2^ *N* ^3^,*O* ^4^)zinc trihydrate

**DOI:** 10.1107/S160053681101676X

**Published:** 2011-05-07

**Authors:** Gang Zhang

**Affiliations:** a Department of Chemistry and Chemical Engineering, Henan University of Urban Construction, Pingdingshan, Henan 467044, People’s Republic of China

## Abstract

In the crystal structure of the title compound, [Zn(C_7_H_7_N_2_O_4_)_2_(H_2_O)_2_]·3H_2_O, the Zn^II^ ion, located an inversion center, is *N*,*O*-chelated by two 5-carb­oxy-2-ethyl-1*H*-imidazole-4-carboxyl­ate anions and further coordinated by two water mol­ecules in a distorted octa­hedral geometry. The carb­oxy group links with the carboxyl­ate group of the same ligand *via* an intra­molecular O—H⋯O hydrogen bond. An extensive inter­molecular N—H⋯O and O—H⋯O hydrogen-bonded network exists in the crystal structure. One disordered lattice water mol­ecule is half-occupied and is located close to an inversion center.

## Related literature

For coordination polymers built from 2-ethyl-4,5-imidazole­dicarboxyl­ate, see: Li *et al.* (2011 ▶); Wang *et al.* (2008 ▶); Zhang *et al.* (2010 ▶).
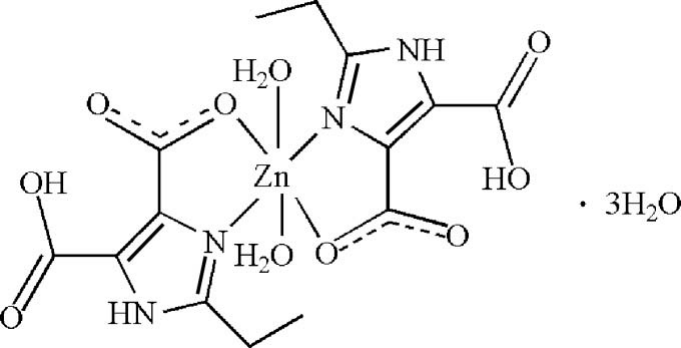


## Experimental

### 

#### Crystal data

[Zn(C_7_H_7_N_2_O_4_)_2_(H_2_O)_2_]·3H_2_O
*M* *_r_* = 521.74
Triclinic, 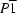

*a* = 7.229 (1) Å
*b* = 8.8959 (12) Å
*c* = 9.3541 (15) Å
α = 65.769 (1)°
β = 88.587 (2)°
γ = 70.676 (1)°
*V* = 513.31 (13) Å^3^
*Z* = 1
Mo *K*α radiation
μ = 1.27 mm^−1^
*T* = 298 K
0.24 × 0.22 × 0.21 mm

#### Data collection

Bruker SMART 1000 CCD area-detector diffractometer
Absorption correction: multi-scan (*SADABS*; Bruker, 2004 ▶) *T*_min_ = 0.750, *T*_max_ = 0.776
2676 measured reflections
1774 independent reflections
1532 reflections with *I* > 2σ(*I*)
*R*_int_ = 0.015

#### Refinement

*R*[*F* ^2^ > 2σ(*F* ^2^)] = 0.041
*wR*(*F* ^2^) = 0.119
*S* = 1.09
1774 reflections
152 parameters
H-atom parameters constrained
Δρ_max_ = 0.45 e Å^−3^
Δρ_min_ = −0.71 e Å^−3^

### 

Data collection: *SMART* (Bruker, 2004 ▶); cell refinement: *SAINT* (Bruker, 2004 ▶); data reduction: *SAINT*; program(s) used to solve structure: *SHELXTL* (Sheldrick, 2008 ▶); program(s) used to refine structure: *SHELXTL*; molecular graphics: *SHELXTL*; software used to prepare material for publication: *SHELXTL*.

## Supplementary Material

Crystal structure: contains datablocks I, global. DOI: 10.1107/S160053681101676X/xu5200sup1.cif

Structure factors: contains datablocks I. DOI: 10.1107/S160053681101676X/xu5200Isup2.hkl

Additional supplementary materials:  crystallographic information; 3D view; checkCIF report

## Figures and Tables

**Table 1:** Selected bond lengths (Å)

Zn1—N1	2.104 (3)
Zn1—O1	2.164 (3)
Zn1—O5	2.116 (3)

**Table 2:** Hydrogen-bond geometry (Å, °)

*D*—H⋯*A*	*D*—H	H⋯*A*	*D*⋯*A*	*D*—H⋯*A*
N2—H2⋯O6^i^	0.86	1.95	2.778 (4)	161
O3—H3⋯O2	0.82	1.65	2.465 (4)	172
O5—H5*C*⋯O3^ii^	0.85	1.95	2.785 (4)	167
O5—H5*D*⋯O4^iii^	0.85	1.88	2.713 (4)	166
O6—H6*E*⋯O4^iv^	0.86	2.29	3.145 (5)	175
O6—H6*F*⋯O7^v^	0.85	2.09	2.664 (17)	125
O7—H7*F*⋯O1^i^	0.85	2.12	2.93 (3)	160
O7—H7*G*⋯O2^ii^	0.85	2.24	3.06 (3)	160

Symmetry codes: (i) 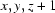; (ii) 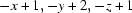; (iii) 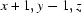; (iv) 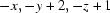; (v) 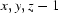.

**Fig. 1.**
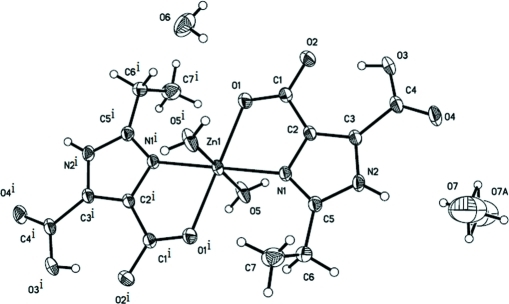
The structure of the title compound, showing the atomic numbering scheme with 30% probability displacement ellipsoids [symmetry codes: i: 1 - x, 1 - y, 1 - z.]

## supplementary crystallographic information

### Comment

Self-assembly of supramolecular architectures based on imidazole carboxylate ligands has draw much attention during recent decades. To the best of our knowledge, coordination polymers based on 2-ethyl-4,5-imidazoledicarboxylate has been rarely reported so far (Wang *et al.*, 2008; Zhang *et al.*, 2010; Li *et al.*, 2011). Herein we report the title compound by the reaction of zinc nitrate with 2-ethyl-4,5-imidazoledicarboxylate (H_3_EIDC) in an aqueous solution under hydrothermal condition.

The title compound, [Zn(C_7_H_7_N_2_O_4_)_2_(H_2_O)_2_].3H_2_O, depicted in Fig. 1, has two symmetrical coordination water molecules, three free water molecules and two 2-ethyl-4,5-imidazoledicarboxylate ligands. the Zn^II^ ion, lying on a center of inversion, is surrounded by two terminal water molecules, two nitrogen atoms and two oxygen atoms from two different 2-ethyl-4,5-imidazoledicarboxylate ligands in a slightly distorted octahedral coordination environment. Three solvent water molecules exist *via* hydrogen bonding among the imidazole N atom, the carboxylate O atom and the O atom from water molecule, whose distances and angles are shown in Tab. 1, Each H_2_EIDC is bonded to Zn^II^ ion in a µ_2_-mode. A three-dimensional supramolecular structure is consolidated by hydrogen-bonding interactions (N—H···O and O—H···O).

### Experimental

A mixture of Zn(NO_3_)_2_ (0.5 mmol, 0.110 g) and 2-ethyl-1*H*-imidazole-4,5-dicarboxylic acid (0.5 mmol, 0.95 g) in an aqueous solution (15 ml) was placed in a 23 ml Teflon-lined reactor, which was heated at 423 K for 2 d, and then cooled to room temperature at a rate of 10 K h^-1^. Crystals of the title compound were obtained by slow evaporation of the solvent at room temperature.

### Refinement

Carboxy H atom was located in a difference map and refined with distance constraint of O—H = 0.82 Å, *U*_iso_(H) = 1.5*U*_eq_(O). Carbon and nitrogen bound H atoms were placed at calculated positions and were treated as riding on the parent C or N atoms with C—H = 0.96 (methyl), 0.97 (methylene) and N—H = 0.86 Å, *U*_iso_(H) = 1.2 or 1.5*U*_eq_(C,N). H atoms of the O6 water molecule were located in a difference Fourier map and refined as riding in as-found relative positions with U_iso_(H) = 1.2U_eq_(O). The O7 atom is located close to an inversion center and is half-occupied in the crystal structure; its H atoms were placed in calculated positions and refined in a riding mode with U_iso_(H) = 1.2U_eq_(O).

### Crystal data

**Table:**

[Zn(C_7_H_7_N_2_O_4_)_2_(H_2_O)_2_]·3H_2_O	*Z* = 1
*M**_r_* = 521.74	*F*(000) = 270
Triclinic, *P*1	*D*_x_ = 1.688 Mg m^−^^3^
Hall symbol: -P 1	Mo *K*α radiation, λ = 0.71073 Å
*a* = 7.229 (1) Å	Cell parameters from 1329 reflections
*b* = 8.8959 (12) Å	θ = 2.4–26.5°
*c* = 9.3541 (15) Å	µ = 1.27 mm^−^^1^
α = 65.769 (1)°	*T* = 298 K
β = 88.587 (2)°	Block, colorless
γ = 70.676 (1)°	0.24 × 0.22 × 0.21 mm
*V* = 513.31 (13) Å^3^	

### Data collection

**Table:**

Bruker SMART 1000 CCD area-detector diffractometer	1774 independent reflections
Radiation source: fine-focus sealed tube	1532 reflections with *I* > 2σ(*I*)
graphite	*R*_int_ = 0.015
φ and ω scans	θ_max_ = 25.0°, θ_min_ = 2.4°
Absorption correction: multi-scan (*SADABS*; Bruker, 2004)	*h* = −8→8
*T*_min_ = 0.750, *T*_max_ = 0.776	*k* = −8→10
2676 measured reflections	*l* = −10→11

### Refinement

**Table:**

Refinement on *F*^2^	Primary atom site location: structure-invariant direct methods
Least-squares matrix: full	Secondary atom site location: difference Fourier map
*R*[*F*^2^ > 2σ(*F*^2^)] = 0.041	Hydrogen site location: inferred from neighbouring sites
*wR*(*F*^2^) = 0.119	H-atom parameters constrained
*S* = 1.09	*w* = 1/[σ^2^(*F*_o_^2^) + (0.0572*P*)^2^ + 0.7163*P*] where *P* = (*F*_o_^2^ + 2*F*_c_^2^)/3
1774 reflections	(Δ/σ)_max_ = 0.001
152 parameters	Δρ_max_ = 0.45 e Å^−^^3^
0 restraints	Δρ_min_ = −0.71 e Å^−^^3^

### Special details

**Table:**

Geometry. All e.s.d.'s (except the e.s.d. in the dihedral angle between two l.s. planes) are estimated using the full covariance matrix. The cell e.s.d.'s are taken into account individually in the estimation of e.s.d.'s in distances, angles and torsion angles; correlations between e.s.d.'s in cell parameters are only used when they are defined by crystal symmetry. An approximate (isotropic) treatment of cell e.s.d.'s is used for estimating e.s.d.'s involving l.s. planes.
Refinement. Refinement of *F*^2^ against ALL reflections. The weighted *R*-factor *wR* and goodness of fit *S* are based on *F*^2^, conventional *R*-factors *R* are based on *F*, with *F* set to zero for negative *F*^2^. The threshold expression of *F*^2^ > σ(*F*^2^) is used only for calculating *R*-factors(gt) *etc*. and is not relevant to the choice of reflections for refinement. *R*-factors based on *F*^2^ are statistically about twice as large as those based on *F*, and *R*- factors based on ALL data will be even larger.

### Fractional atomic coordinates and isotropic or equivalent isotropic displacement parameters (Å^2^)

**Table:**

	*x*	*y*	*z*	*U*_iso_*/*U*_eq_	Occ. (<1)
Zn1	0.5000	0.5000	0.5000	0.0323 (2)	
N1	0.3641 (4)	0.6476 (4)	0.6256 (3)	0.0279 (7)	
N2	0.2272 (4)	0.7940 (4)	0.7637 (4)	0.0323 (7)	
H2	0.1798	0.8155	0.8413	0.039*	
O1	0.4472 (4)	0.7653 (3)	0.3230 (3)	0.0387 (6)	
O2	0.3323 (5)	1.0446 (4)	0.2846 (3)	0.0471 (8)	
O3	0.1810 (4)	1.2103 (3)	0.4395 (3)	0.0459 (7)	
H3	0.2405	1.1501	0.3944	0.069*	
O4	0.0686 (4)	1.1604 (4)	0.6715 (4)	0.0461 (7)	
O5	0.7784 (4)	0.4784 (4)	0.5890 (4)	0.0511 (8)	
H5C	0.8109	0.5636	0.5848	0.061*	
H5D	0.8821	0.3872	0.6159	0.061*	
O6	0.1656 (5)	0.8359 (5)	0.0415 (4)	0.0734 (11)	
H6E	0.1063	0.8394	0.1212	0.088*	
H6F	0.1745	0.9376	−0.0026	0.088*	
O7	0.439 (3)	0.987 (4)	0.987 (3)	0.169 (9)	0.50
H7F	0.4629	0.9037	1.0795	0.202*	0.50
H7G	0.5168	0.9516	0.9288	0.202*	0.50
C1	0.3691 (5)	0.8809 (5)	0.3702 (4)	0.0310 (8)	
C2	0.3209 (5)	0.8245 (4)	0.5336 (4)	0.0274 (8)	
C3	0.2351 (5)	0.9175 (5)	0.6180 (4)	0.0290 (8)	
C4	0.1541 (6)	1.1095 (5)	0.5770 (5)	0.0343 (9)	
C5	0.3068 (5)	0.6320 (5)	0.7650 (4)	0.0304 (8)	
C6	0.3164 (6)	0.4663 (5)	0.9015 (5)	0.0386 (9)	
H6A	0.2978	0.4874	0.9956	0.046*	
H6B	0.4466	0.3783	0.9188	0.046*	
C7	0.1624 (8)	0.3961 (7)	0.8768 (6)	0.0579 (13)	
H7A	0.1874	0.3649	0.7895	0.087*	
H7B	0.0336	0.4849	0.8544	0.087*	
H7C	0.1681	0.2938	0.9706	0.087*	

### Atomic displacement parameters (Å^2^)

**Table:**

	*U*^11^	*U*^22^	*U*^33^	*U*^12^	*U*^13^	*U*^23^
Zn1	0.0374 (4)	0.0234 (3)	0.0368 (4)	−0.0042 (3)	0.0041 (3)	−0.0184 (3)
N1	0.0294 (16)	0.0241 (15)	0.0311 (16)	−0.0060 (12)	0.0031 (12)	−0.0152 (13)
N2	0.0336 (17)	0.0309 (17)	0.0357 (17)	−0.0051 (14)	0.0058 (13)	−0.0222 (14)
O1	0.0490 (17)	0.0309 (14)	0.0351 (15)	−0.0090 (12)	0.0111 (12)	−0.0174 (12)
O2	0.072 (2)	0.0276 (15)	0.0381 (16)	−0.0160 (14)	0.0135 (15)	−0.0120 (13)
O3	0.0598 (19)	0.0240 (14)	0.0548 (19)	−0.0083 (13)	0.0056 (15)	−0.0226 (14)
O4	0.0492 (18)	0.0328 (15)	0.0566 (18)	−0.0010 (13)	0.0059 (14)	−0.0298 (14)
O5	0.0344 (16)	0.0329 (16)	0.090 (2)	−0.0026 (12)	−0.0071 (15)	−0.0364 (17)
O6	0.073 (2)	0.095 (3)	0.055 (2)	−0.014 (2)	0.0122 (18)	−0.047 (2)
O7	0.15 (2)	0.154 (14)	0.125 (11)	−0.022 (16)	0.017 (16)	−0.013 (10)
C1	0.034 (2)	0.0264 (19)	0.034 (2)	−0.0091 (16)	0.0034 (16)	−0.0152 (16)
C2	0.0266 (18)	0.0230 (18)	0.0333 (19)	−0.0067 (14)	−0.0006 (14)	−0.0139 (15)
C3	0.0283 (19)	0.0248 (18)	0.0352 (19)	−0.0064 (15)	0.0006 (15)	−0.0162 (16)
C4	0.032 (2)	0.0264 (19)	0.046 (2)	−0.0056 (16)	−0.0028 (17)	−0.0206 (19)
C5	0.0291 (19)	0.0295 (19)	0.034 (2)	−0.0064 (15)	0.0017 (15)	−0.0170 (16)
C6	0.045 (2)	0.033 (2)	0.033 (2)	−0.0097 (18)	0.0042 (18)	−0.0130 (17)
C7	0.069 (3)	0.058 (3)	0.048 (3)	−0.034 (3)	0.004 (2)	−0.014 (2)

### Geometric parameters (Å, °)

**Table:**

Zn1—N1	2.104 (3)	O5—H5D	0.8499
Zn1—N1^i^	2.104 (3)	O6—H6E	0.8578
Zn1—O1	2.164 (3)	O6—H6F	0.8502
Zn1—O1^i^	2.164 (3)	O7—O7^ii^	1.05 (3)
Zn1—O5	2.116 (3)	O7—H7F	0.8500
Zn1—O5^i^	2.116 (3)	O7—H7G	0.8500
N1—C5	1.324 (5)	C1—C2	1.473 (5)
N1—C2	1.375 (4)	C2—C3	1.366 (5)
N2—C5	1.358 (5)	C3—C4	1.490 (5)
N2—C3	1.369 (5)	C5—C6	1.483 (5)
N2—H2	0.8600	C6—C7	1.509 (6)
O1—C1	1.243 (4)	C6—H6A	0.9700
O2—C1	1.277 (4)	C6—H6B	0.9700
O3—C4	1.286 (5)	C7—H7A	0.9600
O3—H3	0.8200	C7—H7B	0.9600
O4—C4	1.218 (5)	C7—H7C	0.9600
O5—H5C	0.8501		
			
N1—Zn1—N1^i^	180.0	H7F—O7—H7G	108.8
N1—Zn1—O5	88.97 (11)	O1—C1—O2	123.1 (3)
N1^i^—Zn1—O5	91.03 (11)	O1—C1—C2	118.0 (3)
N1—Zn1—O5^i^	91.03 (11)	O2—C1—C2	118.9 (3)
N1^i^—Zn1—O5^i^	88.97 (11)	C3—C2—N1	109.7 (3)
O5—Zn1—O5^i^	180.00 (16)	C3—C2—C1	131.9 (3)
N1—Zn1—O1	78.99 (10)	N1—C2—C1	118.5 (3)
N1^i^—Zn1—O1	101.01 (10)	C2—C3—N2	105.4 (3)
O5—Zn1—O1	91.97 (11)	C2—C3—C4	132.7 (4)
O5^i^—Zn1—O1	88.03 (11)	N2—C3—C4	121.8 (3)
N1—Zn1—O1^i^	101.01 (10)	O4—C4—O3	124.9 (4)
N1^i^—Zn1—O1^i^	78.99 (10)	O4—C4—C3	119.8 (4)
O5—Zn1—O1^i^	88.03 (11)	O3—C4—C3	115.4 (3)
O5^i^—Zn1—O1^i^	91.97 (11)	N1—C5—N2	109.7 (3)
O1—Zn1—O1^i^	180.00 (11)	N1—C5—C6	126.4 (3)
C5—N1—C2	106.7 (3)	N2—C5—C6	123.8 (3)
C5—N1—Zn1	142.7 (3)	C5—C6—C7	112.4 (3)
C2—N1—Zn1	110.7 (2)	C5—C6—H6A	109.1
C5—N2—C3	108.6 (3)	C7—C6—H6A	109.1
C5—N2—H2	125.7	C5—C6—H6B	109.1
C3—N2—H2	125.7	C7—C6—H6B	109.1
C1—O1—Zn1	113.8 (2)	H6A—C6—H6B	107.9
C4—O3—H3	109.5	C6—C7—H7A	109.5
Zn1—O5—H5C	125.6	C6—C7—H7B	109.5
Zn1—O5—H5D	124.4	H7A—C7—H7B	109.5
H5C—O5—H5D	108.7	C6—C7—H7C	109.5
H6E—O6—H6F	102.5	H7A—C7—H7C	109.5
O7^ii^—O7—H7F	88.8	H7B—C7—H7C	109.5
O7^ii^—O7—H7G	79.9		
			
N1^i^—Zn1—N1—C5	55 (100)	O2—C1—C2—C3	1.8 (6)
O5—Zn1—N1—C5	−87.4 (4)	O1—C1—C2—N1	−0.1 (5)
O5^i^—Zn1—N1—C5	92.6 (4)	O2—C1—C2—N1	−178.9 (3)
O1—Zn1—N1—C5	−179.6 (4)	N1—C2—C3—N2	0.2 (4)
O1^i^—Zn1—N1—C5	0.4 (4)	C1—C2—C3—N2	179.6 (4)
N1^i^—Zn1—N1—C2	−127 (100)	N1—C2—C3—C4	−177.8 (4)
O5—Zn1—N1—C2	90.9 (2)	C1—C2—C3—C4	1.5 (7)
O5^i^—Zn1—N1—C2	−89.1 (2)	C5—N2—C3—C2	0.0 (4)
O1—Zn1—N1—C2	−1.3 (2)	C5—N2—C3—C4	178.3 (3)
O1^i^—Zn1—N1—C2	178.7 (2)	C2—C3—C4—O4	173.7 (4)
N1—Zn1—O1—C1	1.4 (3)	N2—C3—C4—O4	−4.1 (5)
N1^i^—Zn1—O1—C1	−178.6 (3)	C2—C3—C4—O3	−5.9 (6)
O5—Zn1—O1—C1	−87.2 (3)	N2—C3—C4—O3	176.3 (3)
O5^i^—Zn1—O1—C1	92.8 (3)	C2—N1—C5—N2	0.4 (4)
O1^i^—Zn1—O1—C1	168 (100)	Zn1—N1—C5—N2	178.7 (3)
Zn1—O1—C1—O2	177.7 (3)	C2—N1—C5—C6	177.6 (3)
Zn1—O1—C1—C2	−1.1 (4)	Zn1—N1—C5—C6	−4.1 (6)
C5—N1—C2—C3	−0.4 (4)	C3—N2—C5—N1	−0.3 (4)
Zn1—N1—C2—C3	−179.3 (2)	C3—N2—C5—C6	−177.5 (3)
C5—N1—C2—C1	−179.8 (3)	N1—C5—C6—C7	−73.4 (5)
Zn1—N1—C2—C1	1.3 (4)	N2—C5—C6—C7	103.4 (4)
O1—C1—C2—C3	−179.4 (4)		

Symmetry codes: (i) −*x*+1, −*y*+1, −*z*+1; (ii) −*x*+1, −*y*+2, −*z*+2.

### Hydrogen-bond geometry (Å, °)

**Table:**

*D*—H···*A*	*D*—H	H···*A*	*D*···*A*	*D*—H···*A*
N2—H2···O6^iii^	0.86	1.95	2.778 (4)	161
O3—H3···O2	0.82	1.65	2.465 (4)	172
O5—H5C···O3^iv^	0.85	1.95	2.785 (4)	167
O5—H5D···O4^v^	0.85	1.88	2.713 (4)	166
O6—H6E···O4^vi^	0.86	2.29	3.145 (5)	175
O6—H6F···O7^vii^	0.85	2.09	2.664 (17)	125
O7—H7F···O1^iii^	0.85	2.12	2.93 (3)	160
O7—H7G···O2^iv^	0.85	2.24	3.06 (3)	160

Symmetry codes: (iii) *x*, *y*, *z*+1; (iv) −*x*+1, −*y*+2, −*z*+1; (v) *x*+1, *y*−1, *z*; (vi) −*x*, −*y*+2, −*z*+1; (vii) *x*, *y*, *z*−1.
